# Screening of a kinase library reveals novel pro-senescence kinases and their common NF-κB-dependent transcriptional program

**DOI:** 10.18632/aging.100845

**Published:** 2015-11-15

**Authors:** Mylène Ferrand, Olivier Kirsh, Audrey Griveau, David Vindrieux, Nadine Martin, Pierre-Antoine Defossez, David Bernard

**Affiliations:** ^1^ Inserm U1052, Centre de Recherche en Cancérologie de Lyon, F-69373 Lyon, France; ^2^ CNRS UMR 5286, F-69373 Lyon, France; ^3^ Centre Léon Bérard, F-69373 Lyon, France; ^4^ Université de Lyon, F-69373 Lyon, France; ^5^ Epigenetics and Cell Fate, University Paris Diderot, Sorbonne Paris Cité, UMR 7216 CNRS, 75013 Paris, France

**Keywords:** senescence, kinases, screen, signaling, NF-κB

## Abstract

Cellular senescence results in proliferation arrest and acquisition of hallmarks such as the Senescence-Associated Secretory Phenotype (SASP). Senescence is involved in regulating numerous physio-pathological responses, including embryonic development, cancer, and several aging-related diseases. Only a few kinases, centered on the RAS signaling pathway, have been identified as inducing premature senescence. About possible other senescence-regulating kinases and signaling pathways, practically little is known. By screening a library of activated kinases, we identified 33 kinases whose constitutive expression decreases cell proliferation and induces expression of senescence markers; p16 and SASP components. Focusing on some kinases showing the strongest pro-senescence effects, we observed that they all induce expression of SASP-component genes through activation of an NF-κB-dependent transcriptional program. Furthermore, inhibition of the p53 or Rb pathway failed to prevent the SASP-inducing effect of pro-senescence kinases. Inhibition of the NF-κB, p53, or Rb pathway proved insufficient to prevent kinase-triggered cell cycle arrest. We have thus identified a repertoire of novel pro-senescence kinases and pathways. These results will open new perspectives in the understanding on the role of cellular senescence in various physio-pathological responses.

## INTRODUCTION

Cellular senescence is a stable form of cell cycle arrest accompanied by acquisition of a Senescence-Associated Secretory Phenotype (SASP), which notably includes production of pro-inflammatory cytokines. Cell senescence participates in various biological processes, including embryonic development, aging-related diseases, wound healing, and tumor protection [1-5].

Activation of cellular senescence in response to aberrant oncogenic activation is a well-described tumor-protective mechanism. In otherwise normal cells, oncogenic activation can trigger cell senescence, blocking the proliferation of potentially defective cells and causing them to develop a SASP that can promote their elimination by the immune system [6]. This mechanism, called Oncogene-Induced Senescence (OIS), is well characterized, and several players involved in it have been identified. Those having attracted the most attention are related to the RAS signaling pathway: oncogenic receptor tyrosine kinases upstream from RAS signaling, MEK kinases in the RAF-MEK-ERK branch downstream of RAS signaling, and the PI3K-AKT kinase branch downstream of RAS signaling have all been found to induce OIS. Despite the importance of these players, the wide range of physio-pathological responses in which cell senescence plays a role suggests that additional signaling pathways and kinases contribute to regulating cell senescence.

To identify cell-senescence-regulating kinases, we have exploited an activated kinase library containing about 200 constitutively active kinases [7]. We show that 33 kinases of this library can induce premature senescence in normal fibroblasts. This suggests that cell senescence can result from activation of diverse signaling pathways, likely to be more numerous than initially believed. We further show that the strongest pro-senescence kinases identified in this screen all induce the SASP via the NF-κB pathway, and that the observed kinase-triggered senescence cannot be alleviated by inhibiting either the Rb or the p53 pro-senescence pathway.

## RESULTS

### A kinase screen identifies new pro-senescence kinases and pathways

**Figure 1:**
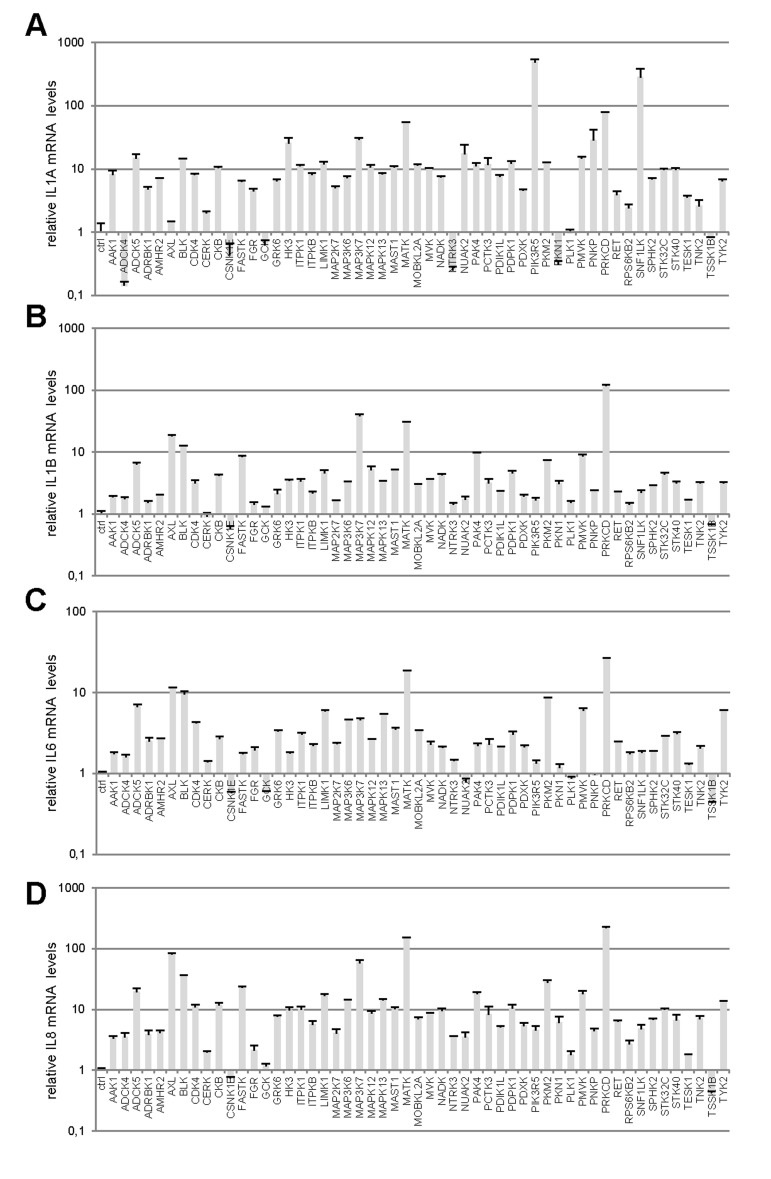

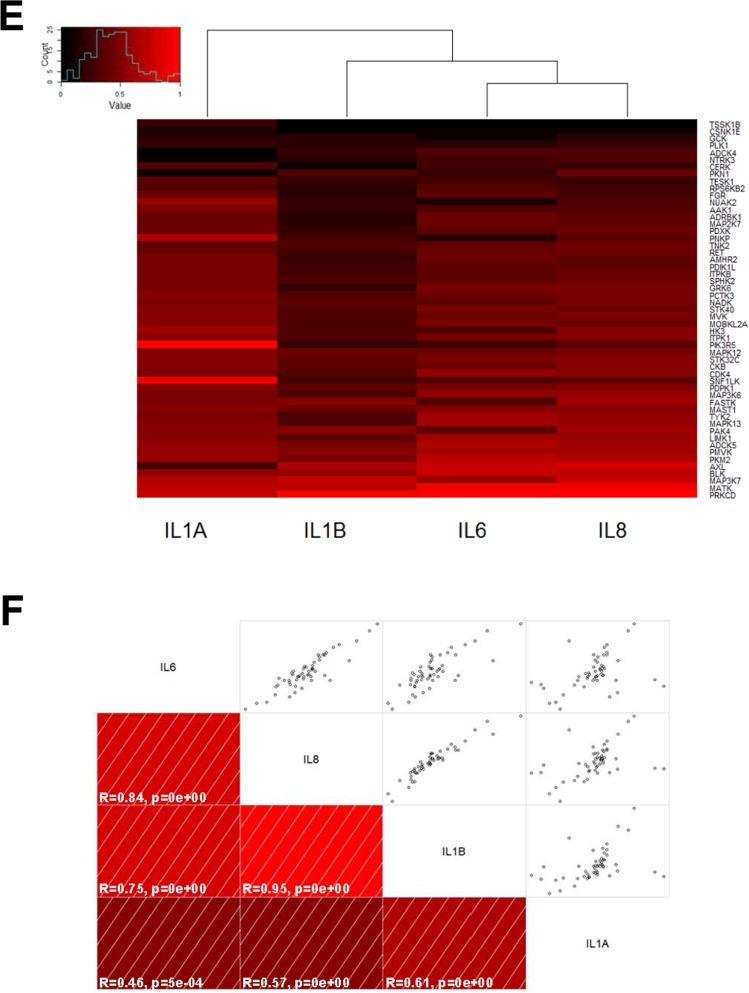
Profiles of SASP component induction by anti-proliferative kinases **(A-D)** IMR-90 normal human fibroblasts were infected with a control vector or the indicated kinase-encoding retroviral vector. Five days after infection, RNAs were prepared and the indicated SASP-component transcript was quantified by RT-qPCR. Results were normalized with respect to the level of ACTB mRNA. **(E)** Relative (log2) fold change induction for each cytokine is represented on a heatmap versus the inducing kinase. **(F)** Correlograms and correlation matrix (non-parametric Spearman method) showing pairwise correlations between fold change induction datasets. Rho values and associated p-values are displayed.

Little is known about the ability of activated kinases other than those acting upstream or downstream from RAS signaling to induce premature senescence. To identify novel cell-senescence-inducing kinases, we stably transduced each kinase gene of a previously described and validated library [7] into normal human fibroblasts (Supplementary Table 1). A first selection on the basis of decreased cell proliferation yielded 53 kinases (Supplementary Table 2). We next examined the effects of these 53 kinases on the levels of transcripts of four well-described SASP components: IL1A (Fig. 1A), IL1B (Fig. 1B), IL6 (Fig. 1C), and IL8 (Fig. 1D) [8,9]. Most of the tested kinases proved able to induce expression of the SASP-component genes, sometimes very strongly (in some cases, more than 100-fold induction was observed) (Fig. 1A-D). Generally, variations in the level of one transcript correlated with changes in the levels of the other three. In others words, the more strongly a kinase induced expression of a SASP-component gene, the more strongly it induced expression of the others (Fig. 1E-F). We found 44 kinases to both decrease cell proliferation and induce expression of SASP-component genes (Supplementary Table 3).

**Figure 2:**
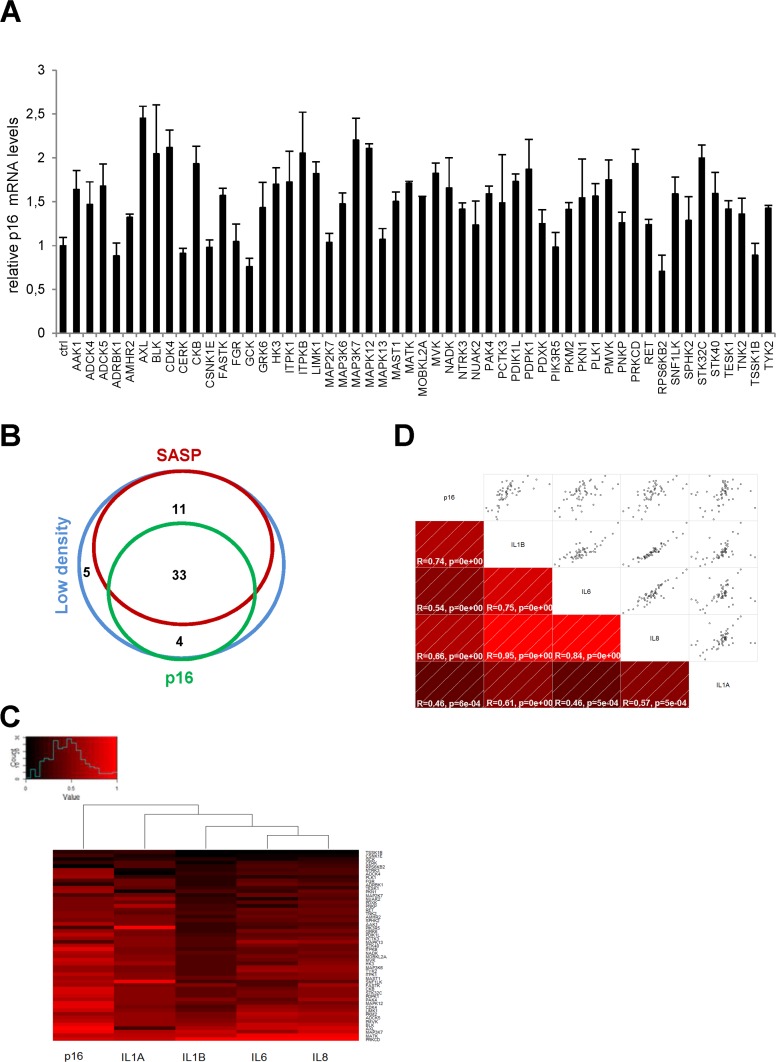
Effects of anti-proliferative kinases on p16 and correlation with SASP-components **(A)** IMR-90 normal human fibroblasts were infected with a control vector or the indicated kinase-encoding retroviral vector. Five days after infection, RNAs were prepared and the p16 transcript was quantified by RT-qPCR. Results were normalized with respect to the level of ACTB mRNA. **(B)** A Venn diagram was drawn to summarize the data. **(C)** For p16 and for each cytokine, relative (log2) fold change induction is represented on a heatmap versus the inducing kinase. **(D)** Correlograms and correlation matrix (non-parametric Spearman method) showing pairwise correlations between fold change induction datasets. Rho values and associated p-values are displayed.

We next focused on p16 transcripts. The p16 protein is a known cyclin-dependent kinase inhibitor (CDKI) whose expression generally increases in senescent cells [10]. The level of p16 transcripts was found to increase in response to numerous kinases, but the fold induction did not exceed about 2.5, in contrast to what was observed with SASP-component transcripts (Fig. 2A). We found 37 kinases both to decrease cell proliferation and to induce p16 gene expression (Supplementary Table 4). As shown in a Venn diagram compiling all the data, we found 33 kinases to decrease cell proliferation and to increase expression of both the p16 gene and the SASP-component genes (Fig. 2B and Table 1). Although the same 33 kinases induced expression of p16 and the SASP, p16 and SASP components appeared in different main branches after hierarchical clustering (Fig. 2C). This suggests that they might be regulated by different transcriptional programs. Still, there was a significant correlation between p16 and the different SASP components (Fig. 2D). A KEGG pathway analysis of these 33 kinases reveals over-representation of some signaling pathways (Table 2) and some of them represent new senescence regulating pathways. In conclusion, we have identified 33 kinases and some associated signaling pathways capable of inducing senescence.

**Table 1:** List of kinases showing decreased cell proliferation and increased p16 and SASP-component induction

AAK1	LIMK1	PCTK3
ADCK5	MAP2K7	PDIK1L
AXL	MAP3K6	PDPK1
BLK	MAP3K7	PIK3R5
CDK4	MAPK12	PKM2
CKB	MAST1	PMVK
FASTK	MATK	PRKCD
GRK6	MOBKL2A	SNF1LK
HK3	MVK	STK32C
ITPK1	NADK	STK40
ITPKB	PAK4	TYK2

**Table 2:** Overrepresented pathways for the 33 pro-senescence kinases

Category	Term	%	PValue	Genes	List Total	Pop Hits	Pop Total	Fold Enrichment
KEGG_PATHWAY	rno04660:T cell receptor signaling pathway	1.36	1.32E-5	MAP3K7, MAPK12, PAK4, PIK3R5, CDK4, MAP2K7	18	109	5590	17.09
KEGG_PATHWAY	rno04930:Type II diabetes mellitus	0.91	3.95E-4	HK3, PKM2, PIK3R5, PRKCD	18	49	5590	25.35
KEGG_PATHWAY	rno04664:Fc epsilon RI signaling pathway	0.91	0.0014	MAPK12, PIK3R5, MAP2K7, PRKCD	18	76	5590	16.34
KEGG_PATHWAY	rno04620:Toll-like receptor signaling pathway	0.91	0.0023	MAP3K7, MAPK12, PIK3R5, MAP2K7	18	90	5590	13.80
KEGG_PATHWAY	rno04722:Neurotrophin signaling pathway	0.91	0.0060	MAPK12, PIK3R5, MAP2K7, PRKCD	18	126	5590	9.86
KEGG_PATHWAY	rno05223:Non-small cell lung cancer	0.68	0.0105	PDPK1, PIK3R5, CDK4	18	52	5590	17.92
KEGG_PATHWAY	rno04012:ErbB signaling pathway	0.68	0.0268	PAK4, PIK3R5, MAP2K7	18	85	5590	10.96
KEGG_PATHWAY	rno04666:Fc gamma R-mediated phagocytosis	0.68	0.0286	LIMK1, PIK3R5, PRKCD	18	88	5590	10.59
KEGG_PATHWAY	rno04912:GnRH signaling pathway	0.68	0.0323	MAPK12, MAP2K7, PRKCD	18	94	5590	9.91
KEGG_PATHWAY	rno00900:Terpenoid backbone biosynthesis	0.453	0.0418	MVK, PMVK	18	14	5590	44.36
KEGG_PATHWAY	rno04010:MAPK signaling pathway	0.91	0.0442	MAP3K7, MAP3K6, MAPK12, MAP2K7	18	266	5590	4.67
KEGG_PATHWAY	rno04910:Insulin signaling pathway	0.68	0.0598	PDPK1, HK3, PIK3R5	18	132	5590	7.06
KEGG_PATHWAY	rno04062:Chemokine signaling pathway	0.68	0.0937	GRK6, PIK3R5, PRKCD	18	171	5590	5.45

### NF-κB pathway activation is a shared characteristic of several pro-senescence kinases

**Figure 3:**
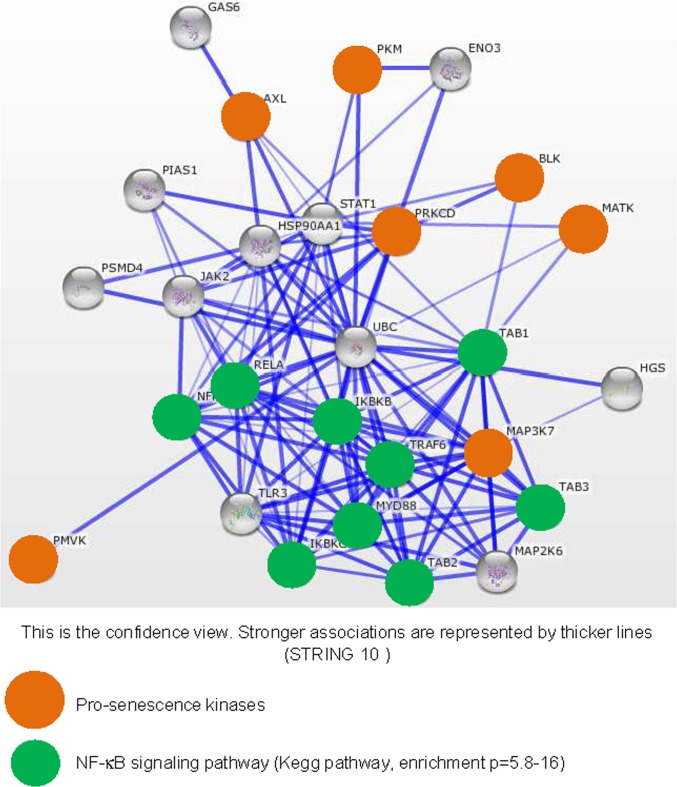
Strongly SASP-inducing kinases display strong associations with the NF-κB signaling pathway The String database (http://string-db.org/) used to build networks on the basis of known and predicted protein-protein interactions was interrogated with, as entries, the indicated pro-senescence kinases (orange circles). Enrichment in KEGG pathways was used to identify common signaling pathways for the pro-senescence kinases. Proteins defined as belonging to the NF-κB signaling pathway are highlighted by green circles.

To see what the identified kinases might have in common, we first interrogated the STRING database to see if at least those displaying the greatest pro-senescence effect, as judged from the level of SASP induction, might share a common protein association network. We built a network based on the seven kinases showing the strongest SASP-inducing effect (Fig. 3). In a KEGG pathway analysis of this network performed to identify overrepresented pathways, we detected strong enrichment in actors of the NF-κB signaling pathway (p=5.8-16) (green circles, Fig. 3). Interestingly, SASP induction during replicative senescence or in response to senescence triggering by the RAS signaling pathway, through RAS or MEK activation, is reported to be mediated by the NF-κB transcription factors [11,12]. These factors are thus good candidate mediators of SASP-component induction by the pro-senescence kinases identified in our screen.

**Figure 4:**
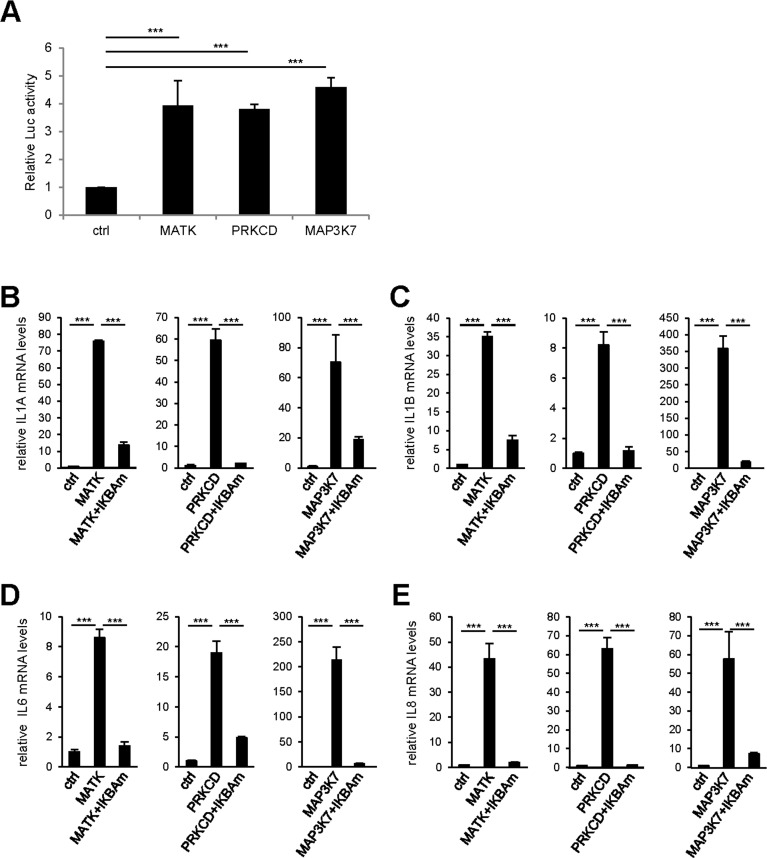
NF-κB activity mediates the effect of pro-senescence kinases on SASP expression **(A)** 293GP cells were co-transfected by the 3κB-Luc reporter and the indicated kinases or the empty vector. Two days later, cells were lysed and Luc activity measured and normalized to protein concentration for each condition. Results are presented as fold Luc activity induced by the indicated kinase when compared to the control vector. **(B-E)** MRC-5 normal human fibroblasts were co-infected with the indicated retroviral vectors. Five days later, RNAs were prepared and RT-qPCR was performed to quantify **(B)** IL1A, **(C)** IL1B, **(D)** IL6, and **(E)** IL8 transcripts. Results were normalized with respect to the level of ACTB mRNA. Statistical analysis was performed with the T-test, p<0.005 ***.

To confirm experimentally the results of this bioinformatic analysis, we first investigated the ability of the three strongest SASP and p16 inducers identified here (Fig. 2C): the MAP3K7, PRKCD, and MATK kinases to activate the NF-κB pathway using a 3κB-Luc reporter vector [13]. The luciferase activity was strongly induced by the 3 kinases indicating that these pro-senescent kinases activate the NF-κB pathway. We next investigated the role of the NF-κB pathway in mediating SASP-component induction by these kinases. We transduced each pro-senescent kinase into human fibroblasts, in combination or not with the gene encoding the IκBα super-repressor (IKBAm), a stabilized NF-κB inhibitor. As expected, the levels of IL1A, IL1B, IL6, and IL8 transcripts strongly increased upon constitutive expression of the kinases (Fig. 4B-E). Strikingly, this induction was almost completely abolished when NF-κB activity was inhibited by IKBAm (Fig. 4B-E). These data demonstrate that SASP component induction by the tested pro-senescence kinases depends on NF-κB transcription factor activity, as anticipated from the STRING bioinformatic analysis.

**Figure 5:**
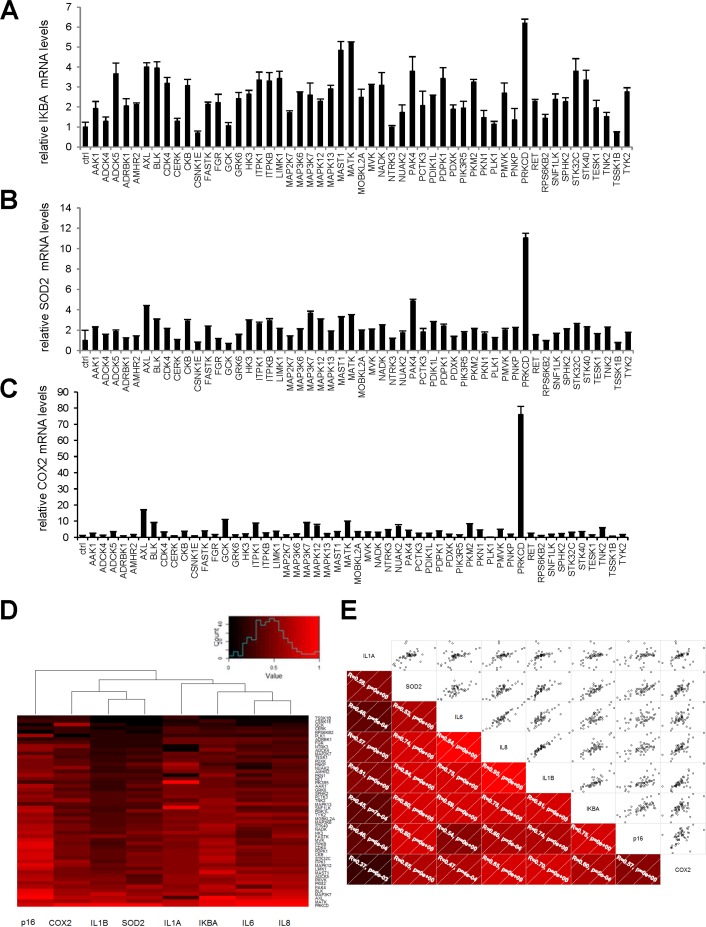
Intracellular NF-κB targets induction by anti-proliferative kinases correlates to SASP components **(A-C)** IMR-90 normal human fibroblasts were infected with a control vector or the indicated kinase-encoding retroviral vector. Five days after infection, RNAs were prepared and RT-qPCR was performed to quantify **(A)** IKBA, **(B)** SOD2, and **(C)** COX2 transcripts. Results were normalized with respect to the level of ACTB mRNA. **(D)** For p16, each cytokine, and each NF-κB target, relative (log2) fold induction is indicated on a heatmap versus the inducing kinase. **(E)** Correlograms and correlation matrix (non-parametric Spearman method) showing correlations between fold change induction datasets. Rho values and associated p-values are displayed.

We next wondered if these pro-senescence kinases might activate a broader NF-κB-dependent program affecting more than just SASP components. To answer this question, we examined the effect of our 33 kinases on transcript-level expression of the genes encoding three known direct intracellular NF-κB targets: IκBα, SOD2, and COX2. The last two genes are known to be involved in NF-κB-induced senescence [14-16]. The kinases were found to induce expression of all three genes (Fig. 5A-C). Upon hierarchical clustering of the SASP components, the intracellular NF-κB targets, and p16 on the basis of their induction profiles, the SASP components and intracellular NF-κB targets were found to cluster on the same main branch, whereas p16 was still on an independent branch (Fig. 5D). This again suggests that expression of the p16 gene is not controlled directly by an NF-κB-dependent transcriptio-nal program. Nevertheless, correlations were observed between p16, individual SASP-component, and individual intracellular NF-κB-target mRNA levels (Fig. 5E).

**Figure 6:**
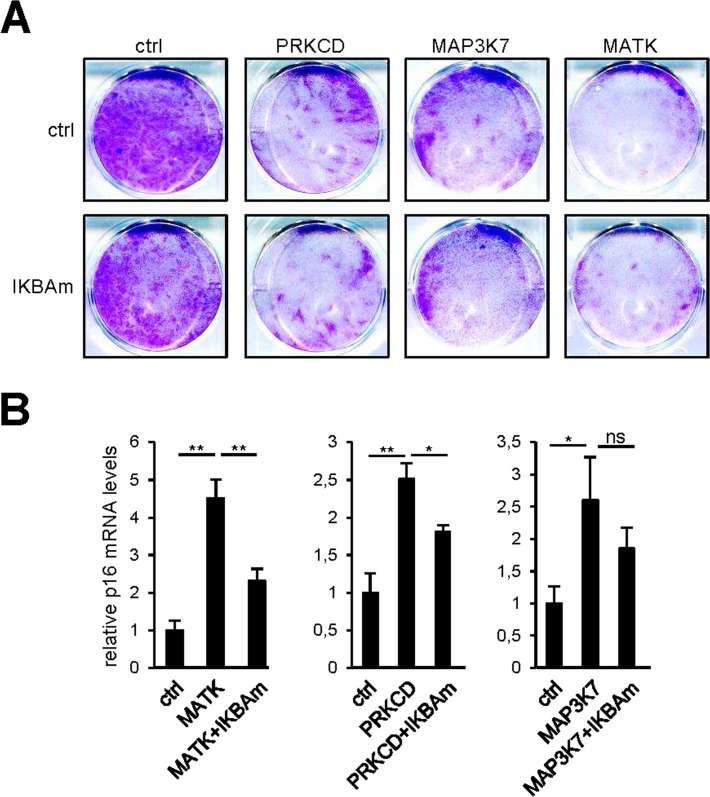
NF-κB target induction is not the sole mediator of senescence by the pro-senescence kinases **(A)** Fifty thousand MRC-5 normal human fibroblasts were seeded per well in 6-well plates. The next day, cells were infected with a retroviral vector encoding the indicated kinase. Ten days after infection, the cells were fixed and crystal violet stained. **(B)** Five days after infection, RNAs were prepared and p16 transcripts were quantified by RT-qPCR. Results were normalized with respect to the level of ACTB mRNA. Statistical analysis was performed with the T-test, p<0.01 **, p<0.05 *.

SASP components can propagate and amplify the senescence signal [8,9,11,17]. COX2 and SOD2 can also promote cellular senescence induced by Rel/NF-κB transcription factors [14-16]. As the responses of all these factors to our pro-senescence kinases appear to be under the transcriptional control of NF-κB, we examined whether constitutive NF-κB inhibition by IKBAm might prevent senescence induction by pro-senescence kinases. As expected, constitutive expression of PRKCD, MATK, or MAP3K7 blocked cell proliferation (Fig. 6A, upper panel) and induced p16 expression (Fig. 6B). Inhibiting NF-κB with IKBAm did not prevent the kinase-promoted proliferation arrest (Fig. 6A) and only partially inhibited p16 induction (Fig. 6B). This suggests that the tested pro-senescence kinases, although strongly inducing transcription of SASP-component and other pro-senescence regulator genes via NF-κB transcription factors, do not induce senescence solely through the NF-κB pathway.

### Inhibition of the p53 or Rb pathway does not prevent senescence or induction of SASP-component gene expression

**Figure 7:**
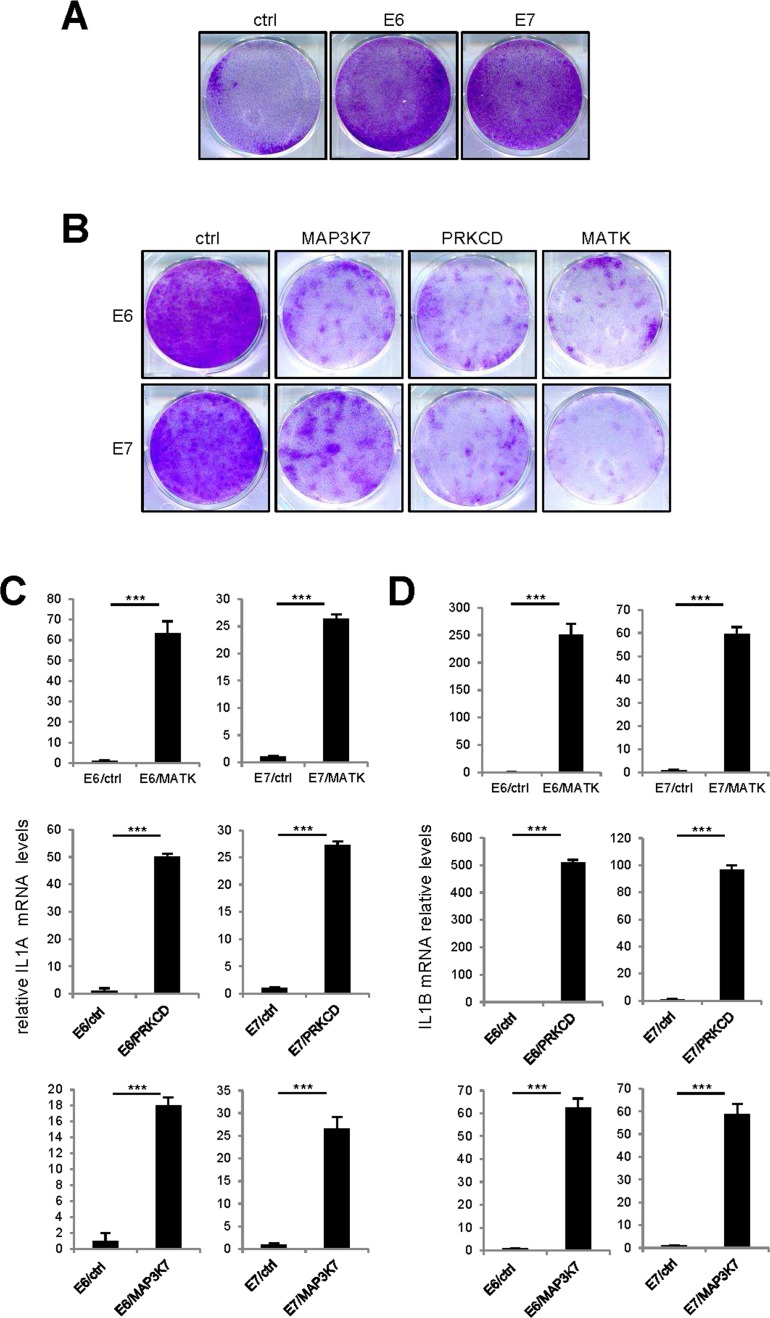

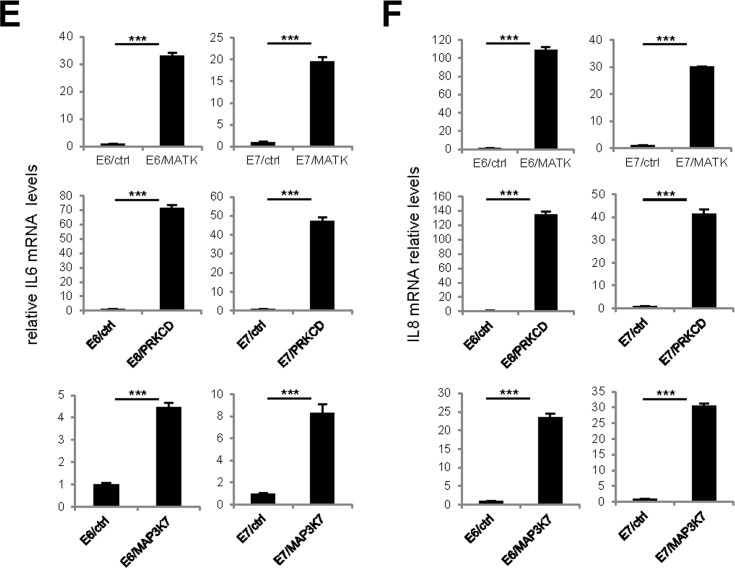
p53 or p16/Rb pathway inhibition did not revert kinase-induced senescence **(A)** One hundred thousand MRC-5 normal human fibroblasts were seeded per well in 6-well plates. The next day, the cells were infected with an E6- or E7-encoding retroviral vector and five days later they were fixed and crystal violet stained. **(B)** Fifty thousand MRC-5 normal human fibroblasts were seeded per well in 6-well plates. The next day, they were infected with the indicated retroviral vector. Thirteen days after infection, the cells were fixed and crystal violet stained. **(C-F)** MRC-5 cells were infected with the indicated retroviral vector and 5 days later, RNAs were prepared. RT-qPCR was performed to quantify **(C)** IL1A, **(D)** IL1B, **(E)** IL6 and **(F)** IL8 transcripts. Results were normalized with respect to the level of ACTB mRNA. Statistical analysis was performed with the T-test, p<0.005 ***.

As the p53 and p16/Rb pathways are important mediators of cell senescence [18,19], we wondered whether they might mediate the cell proliferation arrest and upregulation of SASP-component expression observed in response to pro-senescence kinases. We thus used protein E6 to inhibit the P53 pathway and protein E7 to inhibit the p16/Rb pathway. The ability of E6 or E7 to strongly inhibit its target pathway was demonstrated by acceleration of cell proliferation in normal human fibroblasts infected with an E6- or E7-encoding vector, as compared to cells infected with a control vector (Fig. 7A). Yet even in the presence of E6 or E7, constitutive expression of the MAP3K7, PRKCD, or MATK kinases was still found to promote cell proliferation arrest (Fig. 7B) and increased IL1A, IL1B, IL6, and IL8 transcript levels (Fig. 7C-F). Combination of p53 and NF-kB inhibition has been showed to prevent senescence induced by an oncogenic RAS [12]. We then tested whether MAP3K7-, PRKCD-, or MATK-induced senescence might be overcome by inhibition of p53 by E6 and NF-κB by IKBAm. Inhibition of p53 and NF-κB largely reverted senescence induced by MAP3K7 or PRKCD but had no effect on senescence induced by MATK (Supplemental Figure).

Together, these results demonstrate that inhibition of the p16/Rb or p53 pathway is not sufficient to prevent the proliferation arrest and induction of SASP-component expression triggered by these pro-senescence kinases. Combined inhibition of NF-kB and p53 can revert senescence induced by only some of these pro-senescence kinases.

## DISCUSSION

Despite the growing number of physiological responses in which cellular senescence is known to participate, little is known about the kinases and signaling pathways that induce senescence. Here we have identified 33 pro-senescence kinases, most of which were not previously known to induce senescence. This rather broad range of pro-senescence kinases might reflect the fact that senescence is a cell stress response induced in many situations where cell homeostasis is perturbed.

Our KEGG pathway analysis of these 33 kinases reveals over-representation of expected pathways, such as the “MAPK signaling pathway” itself or other pathways located downstream from receptors that activate it, such as the “T cell receptor signaling pathway”, the “Fc epsilon RI signaling pathway”, and the “Toll-like receptor signaling pathway” (Table 2). Over-representation of a “chemokine signaling pathway” also makes sense, since SASP components, particularly chemokines, are known inducers of cell senescence (Table 2) [9,11,20].

More interestingly, this analysis also reveals new putative pro-senescence pathways. Particularly appealing is the over-representation of the KEGG pathways “Type II diabetes mellitus (T2D)” and “Insulin signaling pathway”, which suggests a possible role of cell senescence in regulating insulin sensitivity and diabetes (Table 2). In line with these results, investigators have linked p16, hallmark of senescent cells, to T2D. For example, T2D susceptibility loci appear associated with the gene encoding p16, and p16 expression increases in tissues derived from diabetic patients. The role of p16 in the etiology of the disease remains unclear, however [21-25,25,26]. The step from these results to demonstrating a role of cell senescence in regulating T2D has not yet been taken, although investigators have begun to discuss this putative role and the possible advantage of targeting senescent cells in order to improve T2D [27,28]. In conclusion, our data support a potential role of cellular senescence in regulating T2D etiology.

The “terpenoid backbone biosynthesis” pathway is also strongly over-represented among our short-listed kinases (Table 2). As far as we know, this pathway has never been implicated in cellular senescence. It leads to production of numerous macromolecules, notably cholesterol, sterol, and ubiquinones. Cellular senescence is thought to be part of a general decrease in health during aging and of the aging process in general [2,27,29]. A decreased MYC level has recently been shown to increase longevity and promote a healthier lifespan in mice [30]. This improvement in the aging process was found to correlate with decreased gene expression in the cholesterol pathway, which is part of the “terpenoid backbone biosynthesis” pathway [30]. Together with our data, these results suggest a possible role for this pathway in promoting cell senescence and aging.

A functional approach on the strongest pro-senescent kinases demonstrates that they all share a common network with NF-κB transcriptional factors and this has been functionally confirmed as NF-κB inhibition is sufficient to block SASP components induction by these kinases. NF-κB is also regulating other intracellular targets showing that senescent cells display a broad chronic NF-κB activation beyond the sole SASP components induction. Focusing on two other pro-senesence pathways, the p53 and p16/Rb pathways, we further demonstrate that inactivation of either one is insufficient to prevent the senescence induced by the identified kinases in normal human fibroblasts. Nevertheless, combined inhibition of p53 and NF-κB is sufficient to reverse senescence by some but not all the pro-senescence kinases tested, suggesting common and distinct pro-senescence pathways induced by the tested kinases.

In conclusion, we have constituted a repertoire of senescence-promoting kinases, including ones not previously known to regulate senescence, and have identified some of the signaling pathways in which they are involved. This repertoire should contribute to establishing and understanding the role cellular senescence plays in the growing list of physio-pathological conditions in which it participates.

## MATERIALS AND METHODS

### Cell culture

IMR-90 (ATCC), MRC-5 (ATCC), and GP293 cells (Clontech) were cultured in DMEM. All media were supplemented with 10% FBS (Sigma) and 1% penicillin/streptomycin (Invitrogen). Upon receipt, cells were thawed and amplified and aliquots frozen. Experiments were performed on the aliquots within a month. The cells were maintained at 37°C under a 5% CO2 atmosphere.

### Vectors, transfection, and infection

The library used was the Myristoylated Kinase Library described in [7] (Kit # 1000000012, Addgene). The retroviral vectors employed were pBabe-Puro-IκBα-mut (super repressor) [7], pLXN-E6, pLXN-E7, and pLXN-E6E7 [31] (Addgene). The 3κB-Luc reporter vector and the protocol for the transactivation assays were previously described [13]. The protocols used to transfect virus-producing GP293 cells and infect target cells have been described previously [32]. The infection protocols were designed so that practically all cells were infected, as judged from the fluorescence observed after infection with a GFP-expressing retroviral vector.

### Colony formation assays

Colony formation assays were carried out in 6-well plates. Depending on the experiment, 50,000 to 100,000 cells were seeded per well. One day later they were infected. Five to ten days after seeding, the cells were washed with PBS, fixed with 4% paraformaldehyde, and stained with 0.05% crystal violet (Sigma-Aldrich).

### RNA extraction, reverse transcription, and PCR

To isolate RNA, we used a phenol-chloroform extraction method involving cell lysis in RNA-ISOL lysis reagent (Dutscher). PhaseLockGel tubes (Prime) were used to separate the phases. Thereafter, the Dynamo cDNA Synthesis Kit (Fisher Scientific) was used for cDNA synthesis from 1 μg total RNA. The RT reaction mixture was diluted 1/20 and the cDNA template used for qPCR analysis. TaqMan quantitative PCR analysis was carried out in the CFX96 Connect Real-Time PCR Detection System (Bio-Rad). The FastStart Essential Probes Master (Roche) was used as PCR mix. The ACTB housekeeping gene was used for normalization. Real-time intron-spanning PCR assays were designed with the ProbeFinder software (Roche Applied Science). The following primers and UPL probes were used: ACTB-Forward ATTGGCAATGAGCGGTTC and ACTB-Reverse GGATGCCACAGGACTCCAT (UPL probe: 11), IL8-F agacagcagagcacacaagc and IL8-R atggttccttccggtggt (UPL probe: 72), IL6-F caggagcccagctatgaac and IL6-R gaaggcagcaggcaacac (UPL probe: 7), IL1A-F ggttgagtttaagccaatcca and IL1A-R tgctgacctaggcttgatga (UPL probe: 6), IL1B-F tacctgtcctgcgtgttgaa and IL1B-R tctttgggtaatttttgggatct (UPL probe: 78), p16-F gtggacctggctgaggag and p16-R ctttcaatcggggatgtctg (UPL probe: 34), IKBA-F gtcaaggagctgcaggagat and IKBA-R atggccaagtgcaggaac (UPL probe: 38), COX2-F gctttatgctgaagccctatga and COX2-R tccaactctgcagacatttcc (UPL probe: 2), SOD2-F aatcaggatccactgcaagg and SOD2-R taagcgtgctcccacacat (UPL probe: 3).

### Heatmaps and correlations

Heatmaps and correlation analyses were done with R (gplots, corrgram, and Hmisc package). Briefly, normalized RT-qPCR tables were loaded in R and relative fold induction was calculated for each cytokine. One in FC induction is set to Zero, maximum FC is set to One (100% induction). Details of calculation are described as supplementary Methods.

## SUPPLEMENTARY MATERIALS AND METHODS, FIGURE AND TABLES


