# Supplementary material for: Screening of a kinase library reveals novel pro-senescence kinases and their common NF-κB-dependent transcriptional program
Source: Aging (Albany NY). 2015 Nov 15;7(11):986–99. doi: 10.18632/aging.100845 (PMC4694068; doi:10.18632/aging.100845)
Supplement: Supplementary file 1 [file aging-07-0986-s001.pdf]

SUPPLEMENTARY MATERIALS AND METHODS

For the different analyses, the macros are as following:

-Heatmap and correlation analyses

```
source("http://bioconductor.org/biocLite.R")
biocLite()
install.packages("gplots")
library("gplots")

#Set Graph title (do one by one)
titre = "allkinases SASP "
titre = "allkinases SASP + p16"
titre = "allkinases SASP + p16 + NFkB"

#Load Normalized RT-QPCR table (do one by one)
allks<-read.table("allkS.txt", header = TRUE, sep = "\t", dec = ",", fill = TRUE, , row.names=1 )
allks<-read.table("allkSp16.txt", header = TRUE, sep = "\t", dec = ",", fill = TRUE, , row.names=1 )
allks<-read.table("allkSp16NFkB.txt", header = TRUE, sep = "\t", dec = ",", fill = TRUE, , row.names=1 )
```

-Log2 relative fold changes induction calculation

```
# matrix of minimun values
min=apply(allks, 2, min )
mat<-matrix(min,
length(row.names(allks)),length(colnames(allks)), byrow=TRUE)
# check
mat

allks2=allks/mat # set minimun of each cytokine to 1
# check
allks2

allks3=log2(allks2)
allks3 # log2 transformation to change 1 to 0


# Matrix of max values
max=apply(allks3, 2, max )
mat2<-matrix(max,
length(row.names(allks)),length(colnames(allks)), byrow=TRUE)

# check
mat2

allks4=allks3/mat2
allks=as.matrix(allks4)
# check
allks
### reorder as minimun to maximum relative induction.
allks2=cbind(allks,apply(allks,1,sum))
allks3=allks2[order(allks2[,length(colnames(allks2))]),]
allks5=allks3[,-length(colnames(allks2))]
# check
allks5
```

-Heatmap

```
#Set a color scale "zero centered"

breaks=c(seq(-(max(allks5)), max(allks5) , by = 0.05))
mycol <- colorpanel(n=length(breaks)-1,low="green",mid="black",high="red")


# Heatmap, "symkey = T"

dev.off()
heatmap.2(allks5, scale = "none", col = mycol, breaks=breaks, symkey = F,
        trace = 'none', cexRow=0.8, cexCol = 1.6, srtCol = 0,
        keysize = 1.3,
        key.title = "",
        margins = c(8, 10),
        main = paste("relative FC"," ", titre),
        Rowv=F
```

-corrgram

```
install.packages("corrgram")

library(corrgram)

corrgram(allks5, order=T, lower.panel=panel.shade,
        upper.panel=panel.pts, text.panel=panel.txt,
        col.regions = colorRampPalette(c("green","black","red")),
        cor.method="spearman",
        main=paste("Correlations"," ", titre))
```

- Rho and pvalue tables

```
install.packages("Hmisc")

library(Hmisc)

rcorr(as.matrix(allks5), type = "spearman")
```

SUPPLEMENTARY TABLES

Table S1. Kinases contained in the kinase library.

AAK1	CMPK	MAP2K7	PFKL	RPS6KA5
ACVR1	CSNK1A1L	MAP3K14	PFKM	RPS6KA6
ADCK4	CSNK1E	MAP3K6	PI4K2B	RPS6KB1
ADCK5	CSNK1G1	MAP3K7	PIK3CB	RPS6KB2
ADPGK	CSNK1G2	MAP3K8	PIK3CG	RPS6KL1
ADRBK1	DAK	MAPK12	PIK3R3	RPSK6A3
ADRBK2	DGKG	MAPK13	PIK3R5	SGK
AKT1	DGUOK	MAPK14	PIK4CA	SNF1LK
AKT3	DLG5	MAPK6	PIK4CB	SPHK2
AMHR2	DYRK2	MAPK7	PIM1	SRPK2
AURKA	DYRK4	MAPKAP1	PIP5K1A	STK17B
AXL	EPHA4	MAST1	PIP5K1B	STK3
BLK	FASTK	MATK	PIP5K2A	STK32A
BMX	FGFR1	MELK	PIP5K3	STK32B
BTK	FGR	MKNK1	PKM2	STK32C
CALM2	FRK	MOBKL1A	PKN1	STK33
CAMK1G	GAK	MOBKL2A	PKN2	STK38L
CAMK2B	GALK2	MPP1	PLAU	STK4
CAMK2D	GCK	MVK	PLK1	STK40
CAMK4	GK	NADK	PLK2	SW1
CAMKK1	GK2	NEK11	PLK3	SW2
CAMKV	GRK5	NEK3	PLK4	SYK
CDC2	GRK6	NEK6	PMVK	TAOK3
CDK2	HCK	NME7	PNKP	TBK1
CDK4	HIPK1	NTRK3	PRKAA1	TEC
CDK5	HK1	NUAK2	PRKACB	TESK1
CDK7	HK2	OXSR1	PRKACG	TIE1
CDK9	HK3	PACSIN1	PRKAG2	TK1
CERK	IHPK2	PAK4	PRKAR2A	TNK2
CHEK1	IKBKE	PAPSS1	PRKCD	TSSK1B
CKB	ILK	PBK	PRKCI	TSSK6
CKM	ITK	PCK2	PRKCZ	TTK
CKMT1A	ITPK1	PCTK1	PRKRA	TYK2
CKMT2	ITPKB	PCTK2	PTK2	UCK2
CKS1B	LCK	PCTK3	RET	ULK4
CKS2	LIMK1	PDIK1L	RIOK1	VRK2
CLK1	LIMK2	PDK1	RIOK2	VRK3

Table S2. List of kinases causing a decreased proliferation.

AAK1	FASTK	MAPK12	PDIK1L	RPS6KB2
ADCK4	FGR	MAPK13	PDPK1	SNF1LK
ADCK5	GCK	MAST1	PDXK	SPHK2
ADRBK1	GRK6	MATK	PIK3R5	STK32C
AMHR2	HK3	MOBKL2A	PKM2	STK40
AXL	ITPK1	MVK	PKN1	TESK1
BLK	ITPKB	NADK	PLK1	TNK2
CDK4	LIMK1	NTRK3	PMVK	TSSK1B
CERK	MAP2K7	NUAK2	PNKP	TYK2
CKB	MAP3K6	PAK4	PRKCD	
CSNK1E	MAP3K7	PCTK3	RET	

Table S2 | IMR-90 cells were infected with a control vector or a vector encoding a kinase of the library (all of the kinases listed in Table S1 were tested). Five days after infection, cell density was examined and only kinases decreasing by at least 50% the cell proliferation were short-listed and presented in this table.

Table S3. List of kinases causing both a cell proliferation decrease and SASP induction (defined as a more than 2-fold increase in the levels of at least two of the four SASP-component gene transcripts).

AAK1	CKB	LIMK1	MATK	PDIK1L	PNKP	TNK2
ADCK5	FASTK	MAP2K7	MOBKL2A	PDPK1	PRKCD	TYK2
ADRBK1	FGR	MAP3K6	MVK	PDXK	RET	
AMHR2	GRK6	MAP3K7	NADK	PIK3R5	SNF1LK	
AXL	HK3	MAPK12	NUAK2	PKM2	SPHK2	
BLK	ITPK1	MAPK13	PAK4	PKN1	STK32C	
CDK4	ITPKB	MAST1	PCTK3	PMVK	STK40	

Table S4. List of kinases causing a decreased cell proliferation and p16 induction (defined as a 1.4-fold or greater increase in p16 transcripts).

AAK1	FASTK	MAP3K7	NTRK3	PKN1	TESK1
ADCK4	GRK6	MAPK12	PAK4	PLK1	TYK2
ADCK5	HK3	MAST1	PCTK3	PMVK	
AXL	ITPK1	MATK	PDIK1L	PRKCD	
BLK	ITPKB	MOBKL2A	PDPK1	SNF1LK	
CDK4	LIMK1	MVK	PIK3R5	STK32C	
CKB	MAP3K6	NADK	PKM2	STK40	

SUPPLEMENTARY FIGURE

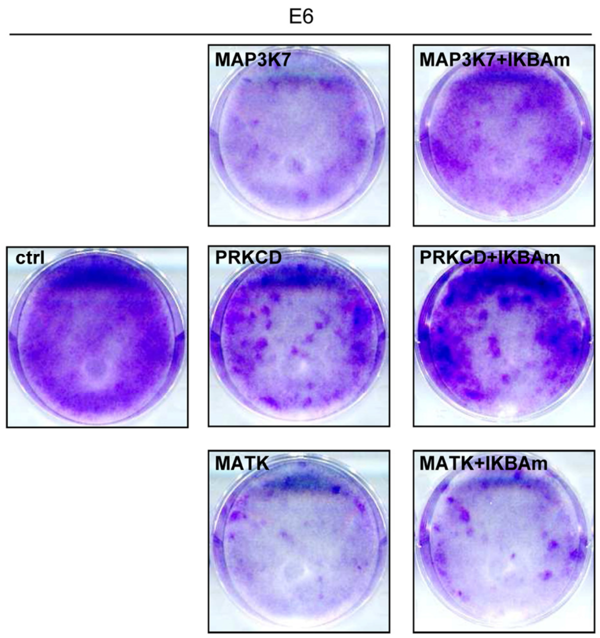

Figure S1. Effect of p53 and NF-kB inhibition on senescence induced by pro-senescent kinases. Fifty thousand MRC-5 normal human fibroblasts were seeded per well in 6-well plates. The next day, the cells were infected with vectors encoding E6, IKBAm and the indicated kinase and 11 days later they were fixed and crystal violet stained.
